# Factors influencing the integration of indigenous and conventional knowledge of water security for livestock

**DOI:** 10.1007/s11250-023-03529-z

**Published:** 2023-03-29

**Authors:** K. Getyengana, E. T. Kamba, M. V. Mkwanazi, S. Z. Ndlela, M. Mwale, M. Chimonyo

**Affiliations:** 1 Animal and Poultry Science, School of Agricultural, Earth and Environmental Sciences, University of KwaZulu-Natal, P Bag X01 Scottsville 3209, Pietermaritzburg, South Africa; 2 Faculty of Science, Engineering and Agriculture, University of Venda, P Bag X5050, Thohoyandou, 0950 South Africa

**Keywords:** Conventional technologies, Indigenous methods, Rituals, Socio-economic status, Water shortages

## Abstract

Farmers have developed indigenous knowledge (IK) on predictive and adaptation strategies to sustain water security. The objective of the study was to determine factors that influence the integration of IK and conventional knowledge (CK) to ensure water security for livestock. Focus group discussions and semi-structured interviews were used to gather data. Farmers in Musina and uMhlabuyalingana use IK indicators to predict rain for water security. Farmers in uMhlabuyalingana predicted rain using wind movement more than their counterparts in Musina (*P* < 0.05). Taboos were used (*P* > 0.05) in both Musina and uMhlabuyalingana to ensure water security. Cultural prohibitions were used more in uMhlabuyalingana to cope with water shortages than in Musina (*P* < 0.05). Boreholes, home taps and municipal water were not properly maintained (*P* > 0.05). Socio-economic factors had greater influence on the integration of IK and CK. Males were 2.24 times likely to support integration of IK and CK compared to women. Adults were 7.1 times likely to support integration of IK and CK compared to those that were younger. Farmers were open to the integration of conventional and indigenous knowledge to ensure water security. Promoting the integration of IK and CK enables stakeholders to gain access to valuable information which can, in turn, promote sustainable community development.

## Introduction

Water insecurity is increasingly becoming a huge challenge for both livestock and humans in developing countries (Mahlangu and Garutsa 2014; Mseleku et al. 2019). Water insecurity, particularly amongst the resource-poor, is caused by the lack of sufficient government support, poor infrastructure, poor maintenance, poor information management and lack of skilled practitioners (Nkuna 2012; Mpendulo et al. 2017). Changes in climates are also exacerbating water shortages. Indigenous knowledge (IK) that have been used for centuries before the introduction of boreholes and piped water have been abandoned. Integrating IK with conventional knowledge (CK) could provide sustainable water systems that benefit both humans and livestock.

Indigenous knowledge refers to long-standing traditions and practices of culturally specific locations. It includes skills, innovations, wisdom, teachings, beliefs and experiences of the people accumulated over years and applied to maintain or improve their livelihood (Vilakazi et al. 2019; Mkwanazi et al. 2021). Indigenous knowledge is rarely found in the mainstream public domain and is not widely taught in schools. Unlike IK, CK is established and is openly supported through government and non-governmental institutions (Maweu 2011), including research. CK is the knowledge that is mostly based on imported knowledge, largely from the Western countries. According to Basdew et al. (2017) conventional knowledge is a formalised process which validates itself using empirical evidence. Conventional methods of conserving rainwater include use of water tanks, installation of household taps and boreholes. CK has failed to sustain water supply for the resource-poor.

Livestock are integral to sustainable livelihoods in most resource-limited communities. They supply meat, milk and other commodities such as skin and mohair (Mapiye et al. 2019). Livestock productivity, however, depends on the availability of feed and water (Ward and McKague 2007). Water availability, therefore, has a direct relationship with food security (Van Emon et al. 2015; Mpendulo et al. 2017). Resource-limited communities get water from rivers, streams and dams. These water systems are usually shared between livestock and humans, resulting in contamination (Nwabor et al. 2016). Water inadequacy has obvious implications for livelihoods. Conventional methods are not sustainable, thus leaving livestock to suffer dehydration and mortality (Basdew et al. 2017).

Despite the struggles and failures of CK to sustainably improve water systems, many resource-poor communities depend on IK for water security. The Intergovernmental Panel on Climate Change (IPCC) has recommended the exploitation of IK in water conservation and management (Petzold et al. 2018). Indigenous methods used for water security include rainmaking ceremonies and water harvesting and use of traditional water ponds. Integration of IK and CK may yield sustainable water security for livestock. For example, communities may possess knowledge on the science of rainmaking, however, lack resources needed to save and store water for longer periods (Limpo et al. 2022; Zvobgo et al. 2022).

Factors that influence the integration of IK and CK in sustaining water security need to be investigated. The integration enables local municipalities and policymakers in sustainable community development processes. It also enriches agricultural extension services in improving water security for both humans and livestock. The objective of the study was to identify factors influencing the integration of IK and CK in ensuring water security. It was hypothesised that integration of indigenous and conventional knowledge varies with socio economic and demographic factors of the household.

## Materials and methods

### Study site

The research was conducted in two district municipalities, uMkhanyakude and Musina. UMkhanyakude is in the northeastern part of KwaZulu-Natal (27°1′ S 32°44′ E) while Musina (22°22′0″ S, 29°45′0″ E) is located in Vhembe District Municipality of Limpopo Province, South Africa. uMhlabuyalingana is a rural area constituting informal settlements and is divided into four traditional council areas (Tembe, Mashabane, Mabaso and Zikhali). The type of natural vegetation found in the municipality is Maputo coastal thicket, growing in a sandy soil. The average annual rainfall is 963 mm (STATSSA 2020). The maximum and minimum temperature varies between 26.3 and 18 °C with an annual average temperature of 22.7 °C, respectively.

The type of vegetation found in Musina municipality is the Bushveld comprising of low-shrub and thorny trees. The type of soil is mostly sandy. Musina receives an annual rainfall of 250 mm, with most rainfall occurring mainly during mid-summer. The highest amount of rainfall (55 mm) is received in January when ambient temperatures average 32.1 °C. The region is coldest during July when the mercury drops to 7.6 °C on average (STATSSA 2020). Musina and uMhlabuyalingana have water security challenges, occurrence of drought as reported by Dzama and Marandure (2016) and availability of livestock.

Farmers in both Musina and uMhlabuyalingana have extensive experience of IK in coping and adapting to climate change trends on water security issues. These two study communities are representative as they shared common experiences with all other indigenous farmers. Both uMhlabuyalingana and Musina communities are resource-poor and have a rich heritage and extensively use IK for survival. Their readiness to integrate the IK and CK is likely to differ due to cultural differences.

### Household selection

uMhlabuyalingana and Musina were chosen because of their experience with water challenges for both humans and livestock. A purposive sample technique was employed to choose participants. Villages in both municipalities were selected based on livestock ownership, lack of access to perennial water supplies and extreme water shortages. Communities that actively participated in the study were Ndlondlweni, Mseleni, KwaMabasa and KwaSonto in uMhlabuyalingana Local Municipality. Domboni and Malale were the two communities from Musina Local Municipality. Scheduled meetings with chiefs, traditional healers, livestock extension officers and local headmen were arranged to gain access to all communities. The data were collected between August and October 2019.

### Focus group discussions

Data were first sourced through interactive focus group discussions (FGD). In each municipality, four FGD were used. Each focus group was made up of between 8 and 15 participants. The groups differed according to age and gender. The first group comprised of young males < 25 years; the second group had females < 25 years; the third group had adult males > 25 years; and the fourth group had females > 25 years. During discussions, information relating to perceptions toward integration of IK and CK in ensuring water security was sought. Structured guidelines were used during the discussions; however, participants were not strictly limited to the guide.

### Administration of structured questionnaire

The interviews targeted 200 households per municipality with a total of 25 respondents randomly selected from each village. A total of 154 households in uMhlabuyalingana and 130 in Musina participated in the study. Households were selected based on the willingness to participate in the study. During the collection of data, enumerators were sourced from the local villages. The researchers trained the enumerators. To ensure that farmers are comfortable to co-operate during the study, local community leaders identified the enumerators. Since the data was collected in two different provinces, questionnaires were presented in the vernacular languages, IsiZulu in uMhlabuyalingana and Tshivenda in Musina.

The questionnaire captured information on household socio-economics, mitigation and adaptation strategies used to ensure human and livestock water security and factors influencing integrating IK and CK. Respondents below the age of 40 years were classified as young adults, middle-aged adults as those between 40 and 59 and the elderly (above 60 years). Professionals were those that have knowledge-based contemporary occupations with skills that were acquired from formal training or education.

### Statistical analyses

Data were analysed using Statistical Analysis System (SAS) (2019). The PROC FREQ procedure for chi-square was used to compute the association between demographics and integration of IK and CK. The FGDs were analysed using themes identified from word‐based techniques, namely, word repetition, indigenous categories and similarities and differences (Ryan and Bernard 2003). Words occurring often are seen as salient in the minds of respondents and useful in understanding what people say (Ryan and Bernard 2003). The PROC GLM procedure (Statistical Analysis System (SAS) 2019) was used to generate least square means to analyse ranks of the indigenous and conventional coping and adaptive strategies employed by farmers to secure water. Ordinal logistic regression was performed using PROC LOGISTIC to predict the odds ratios of the likelihood of integrating IK and CK methods to ensure water security. The dependent variable was either yes or no to whether farmers integrated IK and CK to secure water. The variables fitted in the model were site, age, gender, level of educational status, employment status, socio-economic status and livestock herd size. The model used was:

$$\mathrm{Ln}\left[P/1-P\right]={\beta }_{0}+{\beta }_{1}{X}_{1}+{\beta }_{2}{X}_{2}+{\beta }_{3}{X}_{3}+{\beta }_{t}{X}_{t}+\varepsilon$$

where:

*P* is the probability of the group integrating IK and CK.

[*P*/1 − *P*] is the odds of the group integrating IK and CK.

*β*_0_ is the intercept.

*β*_1_…*β*t are the regression coefficients of predictors.

*X*_1_…*X*_t_ are the predictor variables.ε is the random residual error.

When computed for each predictor (β1…βt), the odds ratio for the group integrates IK and CK.

## Results

### Focus group discussions

The group discussions focused on accurately determining the feed used for livestock during water scarcity, indigenous predictive and coping strategies used, water source management and conventional methods employed to store water during drought (Table 1). Farmers use commercial feed, natural pastures and crop residues in both uMhlabuyalingana and Musina. Again, farmers build earth dams so that they can have enough water for livestock (Table 1). Other farmers often harvest rainwater and store it for dry seasons and during drought.

**Table 1:** Perspectives of different FGDs in both uMhlabuyalingana and Musina area

Theme	Description
Feed in water shortages for cattle	Commercial feed, natural pastures, crop residues, natural pastures and commercial feed combined
Indigenous coping strategies	Transhumance, rituals, taboos, cultural prohibitions
Indigenous predictive strategies	Bio-indicator, astrology, weather trends
Water source management	Rainwater harvest, earth dams
Methods adopted from conventional knowledge	Tanks, boreholes, water tanker trucks

Farmers with no boreholes in their homesteads used tanks to harvest rainwater from the roof. Farmers used indigenous coping strategies such as performing rituals to appease the ancestors, transhumance to nearby non-drought affected areas, taboos and cultural prohibitions. Farmers also revealed that they use weather trends and bio-indicators (birds, animal behaviour) to predict drought. In addition, they also depend on astrological indicators to predict the occurrence of drought.

### Indigenous predictive strategies used to ensure water security

Table 2 shows indigenous strategies used to ensure water security. The use of bird behaviour as a predictive strategy was not different (*p* > 0.05). The use of insects to predict rain was no different (*p* > 0.05). In uMhlabuyalingana, farmers observed insects more than farmers in Musina to predict drought. Cloud patterns and the shape of the moon and stars were used to predict rain in both areas were different (*p* < 0.05). Farmers in Musina, however, observed moon shapes more than farmers in uMhlabuyalingana.

**Table 2:** Predictive strategies adopted to ensure water security

Methods	uMhlabuyalingana	Musina	*P* value
Bio-indicators			
Bird behaviour	1.7	1.6	NS
Insect indicators	1.5	1.2	**
Astrology			
Cloud patterns	1.6	1.5	*
Moon shape	2.2	4.0	*
Stars	1.58	1.11	***
Weather trends			
Wind movement	6.04	5.18	*
Mist-covered mountains	3.14	2.94	NS
Black ants	2.7	2.9	NS

*NS* not significant (*P* > 0.05); *P < 0.05; **P < 0.01; ***P < 0.001

Values are expressed as least square means with lower values being the highest and higher values the least

Stars were used more by farmers in uMhlabuyalingana than those in Musina in prediction of drought (Table 2). There were significant differences in the use of black ants and mist-covered mountains to predict rain between the two areas. The use of wind movement was also associated (*p* < 0.05) with site. Farmers in uMhlabuyalingana predicted rain using wind movement more than their counterparts in Musina.

### Indigenous coping strategies used during water scarcity

Strategies that farmers adopted to cope with water security are shown in Table 3. There was no association (*p* > 0.05) between uMhlabuyalingana and Musina and the use of transhumance to cope with water challenges. Farmers in uMhlabuyalingana were performing rituals more (*p* < 0.05) than farmers in Musina to appease ancestors as a means of pleading for rain. Elderly men and chiefs (*Izinduna*) and kings (*Amakhosi*) visit forest, caves and mountain peaks with traditional beers and gifts to appease ancestors for water. If the processes are handled well, then rains occur, as expected. The rainmaking ceremonies are highly confidential and only selected few individuals participate. There was no association between the uMhlabuyalingana and Musina and the use of taboos (*p* > 0.05). Cultural prohibitions were used more in uMhlabuyalingana to cope with water shortages than in Musina (*p* < 0.05).

**Table 3:** Coping strategies adopted for water security

Method	uMhlabuyalingana	Musina	*P* value
Transhumance	1.5	1.0	NS
Rituals	1.5	1.2	**
Taboos	3.14	2.94	NS
Cultural prohibitions	1.58	1.11	**

*NS* not significant (*P* > 0.05); **P < 0.01

Values are expressed as least square means with lower values being the highest and higher values the least

### Conventional methods used to ensure water security

Farmers in uMhlabuyalingana were depending (*p* < 0.05) on rainwater harvesting more than farmers in Musina. Other methods used to ensure water security were wells and dams (Table 4). The use of wells to ensure water security was more prevalent in uMhlabuyalingana than in Musina. Additionally, dams were used more in uMhlabuyalingana compared to Musina (*p* < 0.05). There was no association between site the use of boreholes and home taps (*p* > 0.05). The use of water tanks to secure water was common (*p* < 0.05). Farmers in Musina depended on water tanks more than those in uMhlabuyalingana.

**Table 4:** Indigenous and conventional strategies employed by farmers to secure water

	Methods used	Areas		*P* value
		uMhlabuyalingana	Musina	
*Indigenous knowledge*				
	Rainwater harvest	4.08	3.20	**
	Wells	3.67	2.4	*
	Dams	1.5	1.2	*
	Rivers	2.0	2.0	NS
*Conventional knowledge*				
	Boreholes	2.10	1.96	NS
	Home taps	3.65	3.27	NS
	Municipal water tanker trucks	2.28	2.36	NS
	Water tanks	2.74	3.73	*

*NS* not significant (*P* > 0.05); *P < 0.05; **P < 0.01

Values are expressed as least square means with lower values being the highest and higher values the least

### Factors affecting integration of indigenous and conventional knowledge

The association between the socio-demographic information of respondents and integration between IK and CK is shown in Table 5. There was an association between gender and integration of IK and CK to ensure water security amongst the participants (*p* < 0.05). Women supported the integration of IK and CK more (60%) than men. There was a significant association between the age of farmers and the integration of IK and CK. Adults (62%) supported the integration of IK and CK more than the youth. There was no association between livestock training, sources of income and integration of IK and CK (*p* > 0.05).

**Table 5:** Household demographics and association with the integration of IK and CK

Profiles	Integration of IK and CK	Chi-square	*P* value
Gender (%)			
Men	39.9	2.06	*
Women	60.1		
Age distribution (%)			
Youths	37.9	4.48	**
Adults	62.1		
Livestock training (%)			
Yes	52.6	1.71	NS
No	47.4		
Employment status (%)			
Employed	25.0	6.51	**
Unemployed	75.0		
Sources of income (%)			
Government grant	51.1		
Salary	9.80	1.25	NS
Livestock products	22.7		
Crops	16.4		
Educational status (%)			
Formal	35.7	9.13	**
Informal	64.3		
Socio-economic status (%)^1^			
Poor	12.6		
Less poor	34.6	3.06	*
Very poor	52.6		

*NS* not significant (*P* > 0.05); *P < 0.05; **P < 0.01

^1^Poor farmers were those with some reliable sources of income, at least 2 cows and owning other forms of livestock. Less poor households had at one household member with a formal employment or have large livestock herds/flocks. Very poor farmers consisted of farmers with few goats, chickens and meagre amounts of income

There was a significant association between employment status and integration of IK and CK. Respondents that were unemployed supported the integration of IK and CK more than (75%) those that are employed. There was an association (*p* < 0.05) between the educational status of respondents and integration of IK and CK. Farmers that had low levels of formal education supported the integration of IK and CK more (64%). The association between socio-economic status of farmers and integration of IK and CK was significant. Farmers that were categorised as very poor supported the integration of IK and CK more than (53%) farmers in other categories.

Table 6 shows odds ratio estimates of factors influencing integration of IK and CK to ensure water security amongst the farmers. There was a highly significant difference between Musina and uMhlabuyalingana on the integration of IK and CK. As a result, the influence of the demographic factors in uMhlabuyalingana and Musina was presented separately. Male farmers in uMhlabuyalingana (*p* < 0.05) were 2.24 times likely to favour the integration of IK and CK to ensure water security. Odds ratio of adults favouring the integration of IK and CK was 7.1 times less than the young people (*p* < 0.05) in uMhlabuyalingana. In uMhlabuyalingana, farmers with large herds were 16.8 times more likely to favour the integration of IK and CK than those with small herds. In Musina, farmers with large cattle herds were 3.47 times more willing to integrate IK with CK than those with small herds. In uMhlabuyalingana, the odds ratio estimates were 16.8 times to favour integration of IK and CK (Table 6). In Musina, however, farmers that are educated were 1.27 more times to favour the integration of the two knowledge systems (*p* < 0.05). The odds ratio estimates of unemployed farmers to favour integration of IK and CK were 1.98 times more than that of employed farmers in uMhlabuyalingana. Farmers classified as poor were 1.67 times more likely to support the integration of IK and CK to ensure water security in Musina (*p* < 0.05).

**Table 6:** Odds ratio estimates and lower and upper confidence interval (CI) for factors influencing integration between IK and CK

Predictor	uMhlabuyalingana				Musina			
	Odds ratio	LCI	UCI	*P* value	Odds ratio	LCI	UCI	*P* value
Gender (male vs female))	2.24	0.41	2.74	*	2.16	0.81	5.79	NS
Age (young vs adults)	0.14	0.03	0.52	*	1.15	0.47	2.79	NS
Education (educated vs uneducated)	0.51	0.13	2.02	NS	4.33	1.27	14.8	*
Employment (employed vs unemployed)	0.53	0.53	12.1	*	1.57	0.43	5.75	NS
Livestock herd (large > 20 versus small ≤ 20)	16.8	2.75	103.4	*	3.47	1.10	10.9	*
Socio-economic status (poor vs. less poor)	0.42	0.36	5.75	NS	1.67	0.36	2.75	*

Higher odds ratio estimates indicate greater difference in occurrence between levels of predictors; *P < 0.05; **P < 0.01; *NS* Not different (*P* > 0.05) vs. indicates versus

## Discussion

Climate change is expected to intensify in the future with severe risks projected for humans and livestock due to sporadic and unreliable rainfall (Basdew et al. 2017). It is, thus, crucial that adaptation actions are implemented, and their effectiveness for reducing risk is investigated. Such adaptive responses include integration of IK and CK. A better understanding of factors influencing integration of IK and CK is crucial in promoting successful strategies of adaptation to ensure that livestock have adequate water. The observation that a larger proportion of women supported the integration of IK and CK was expected. Many households in communal production systems are female-headed because of high migration of men to urban areas in search of employment opportunities (Vilakazi et al. 2019; Ndlela et al. 2021). Thus, women remain behind to look after the livestock and children with little remittances that are not sufficient for them to install water systems, such as boreholes, for secure water for them and livestock. Most women are also over-burdened with wide range of activities, tasks and responsibilities in agriculture and animal production (Ncobela and Chimonyo 2015). Thus, integration of IK skills and CK infrastructure is likely to prevent women from travelling long distances in search for water.

The finding that the elderly were in favour of integrating IK and CK could be explained by the strong attachment that adults have with their culture. Most of the farmers with large cattle herds were also elderly. The youths due to widespread availability of cell phones find it easy and convenient to search for information, which improves livestock. Most of the available knowledge is conventional. The younger generations are, therefore, more receptive to CK than IK (Ndlela et al. 2021; Bulcha et al. 2022). Most youths associate IK with backwardness. The lack of interest in IK by the youths could also be attributed to the conventional schooling system that does not incorporate IK in its syllabi (Weckmüller et al. 2019). In most cases, IK is despised. It is also important to ensure that communities are motivated to undertake cultural obligations towards achieving water security (Mahlangu and Garutsa 2014).

As expected, most unemployed farmers supported the integration of IK and CK. A combination of the two knowledge systems is expected to equip farmers with knowledge and skills to adapt to changing climates and improve agricultural productivity, profitability and livelihoods. The supply of fodder from extension services assists farmers not to only depend on natural pastures for feeding their livestock (Fakade 2016). Low and erratic rainfall leads to drying out of pastures. The exploitation of indigenous coping and predictive strategies of droughts in resource-limited farmers has been reported earlier. Indigenous knowledge provides substantial contributions toward addressing the issues of water security and mitigation of natural disasters (Borthakur and Singh 2020). Indigenous water coping systems have been used in many parts of the world from time immemorial (Behailu et al. 2016).

The finding that behaviour of birds was used in the prediction of drought agrees with Vilakazi et al. (2019) who reported that through long-term observations farmers have studied the behaviour of birds, wind and cloud patterns amongst other indigenous indicators to predict weather. Farmers also used sounds of bees, spiders and frogs to predict rain in uMhlabuyalingana, highlighting their strong dependence on IK. When spiders move down from their web, it is indicative of rain. When frogs are heard singing in the early evening, it signifies the coming of fair weather on the next day (Kalanda-Joshua et al. 2011). Due to the long-time use of IK, some farmers are not easily willing to work with imported techniques (Limpo et al. 2022). Bird behaviour has been used from time immemorial as indicators of rain. When birds sing with a sharp voice, it indicates that rain is surely coming (Luseno et al. 2003). The use of IK prediction methods is, therefore, convenient and provides clues to farmers about reducing risk (Mafongoya and Ajayi 2017). The use of cloud patterns, moon shape, black ants and wind patterns to predict drought for water security in both study areas agrees with Basdew et al. (2017). Through long-term observations of weather, farmers have experience to relate cloud patterns with the prediction of rain (Vilakazi et al. 2019). In many African cultures, wind speed and direction are also used as a predictor of rainfall (Mafongoya and Ajayi 2017). Warm fast-moving winds during the hot dry season indicate that the upcoming rainy season is short or that the rainfall will be erratic. Winds blowing from west to east are likely to suggest the onset of the rainy season (Vilakazi et al. 2019). More farmers in uMhlabuyalingana predicted rain using wind movement more than their counterparts in Musina. This could be influenced by the geographical location of uMhlabuyalingana and Musina. uMhlabuyalingana is situated in coastal areas that are windier than the inland. As such, the speed and direction of the wind have more value in predicting rainfall patterns in uMhlabuyalingana than in inland Musina environment which are usually calm for most parts of the year (Elia et al. 2014).

When the moon is half-shaped, farmers predict that it will rain in the coming days (Borthakur and Singh 2020). Zuma-Netshiukhwi et al. (2013) reported that, amongst the Sotho communities, a moon facing upwards means upholding water while facing downwards represents that there will be rain in the next three days. It was surprising that transhumance was no longer a common practice in both uMhlabuyalingana and Musina. This could have been influenced by the difference in climate between the two study sites; the rainfall is much higher in uMhlabuyalingana, thus making fodder more available for cattle than in Musina. It is a common thing that during droughts, farmers move their livestock to neighbouring villages to mitigate the effects of drought. Thus, a probable explanation for the failure to practice transhumance in both Musina and uMhlabuyalingana could be fear of livestock theft (Chakoma et al. 2016; Matope et al. 2020). It is imperative to disseminate IK to assist farmers in the prediction of impending droughts to give them opportunities to adopt IK technologies (Mavhura et al. 2015).

The observation that farmers in uMhlabuyalingana performed rituals more than farmers in Musina to appease ancestors as a means of pleading for rain concurs with Mahlangu and Garutsa (2014). Ancestors are considered as guides and mentors that supply people with rain. It is not clear why such ceremonies are not as common in Musina as in uMhlabuyalingana. The integration of CK and IK could suggest an increased frequency of holding rituals. It is, thus, necessary that such rituals are acknowledged, appreciated and made known to all community members. Rainmaking ceremonies are now rare, which could have made ancestors angry (Basdew et al. 2017). Thus, there need to revive such ceremonies to mitigate and adapt to water challenges for the future.

The higher prevalence of cultural prohibitions in uMhlabuyalingana than Musina highlights regional differences on the reliance on IK. The high dependence on rainwater harvesting by farmers in uMhlabuyalingana could be due to the severity of droughts in the area. Rainwater harvesting is one of the common indigenous conservation and water management methods (Grey and Sadoff 2007). Mahlangu and Garutsa (2014) showed that nearly 76% of farmers in rural areas harvest water for pasture improvement and livestock drinking. Wells and dams are important and effective water points, especially in low rainfall regions (Mahlangu and Garutsa 2014).

The use of boreholes, home taps and municipal water trucks could be convenient for farmers to access water. Such conventional methods, however, require high capital investments, which are hardly available to rural farmers. The higher likelihood of men to prefer the integration of IK and CK highlights the dominance of the patriarchal decision structure in uMhlabuyalingana (Ndlela et al. 2021). Both Basdew et al. (2017) and Mkwanazi et al. (2020) observed that women are less likely to act on weather and climate information than men.

The observations that adult farmers in uMhlabuyalingana were in favour of integrating IK and CK in ensuring water security could be an indication of the desire to ensure sustainable water security. Adults support families through farming, while young people mostly do not value IK. As such, most adults wish that IK be disseminated and taught to the youths as well, even in schools (Ladislaus et al. 2010). The finding that educated farmers supported the integration of IK and CK in Musina could be influenced by the high literacy levels amongst the VhaVenda people in South Africa. During discussions, the locals in Musina felt that farmers would benefit from the integration of IK and CK as they will be able to use both systems to solve water security challenges (Rukema and Simelane 2013). The finding that unemployed farmers in uMhlabuyalingana favoured the integration of IK and CK to ensure water security highlights the possible role of integration in creating employment opportunities and creating vibrant rural economies. The findings that households with large herd sizes favoured the integration of IK and CK could reflect the higher need for a sustainable water source than when herd sizes are small.

## Conclusions

The integration of IK and CK greatly contributes towards security to water. Indigenous knowledge was favoured in both uMhlabuyalingana and Musina to ensure water security for both humans and livestock. The integration of IK and CK is influenced by socio-economic and household demographic factors. These factors should be considered in formulating policies that promote sustainable water security for livestock and humans. There is need to create platforms that ensures that the integration of these knowledge systems get implemented.

## Data Availability

Data will be made available upon request.
